# Recurring Jaundice in Successive Pregnancies

**Published:** 1914-05-23

**Authors:** 


### Recurring Jaundice in Successive Pregnancies.

In the *Medical Chronicle* Dr. F. E. Tylecote publishes notes of an exceedingly curious case, only one previous instance of which is on record (Dr. H. D. Rolleston's case, 1910). The features of this rare condition are recurring jaundice in consecutive pregnancies, together with fatal jaundice in the babies. The patient was married at eighteen years of age and has had eight children. They were all born prematurely, as in the case reported by Dr. Rolleston. In each pregnancy the mother became jaundiced at about the third month, to a degree increasing right up to delivery. After her confinements the jaundice disappeared in two or three months as a rule; but since the last one, six years ago, it has never left her at all. She never had any pain with the jaundice, nor was there hyperemesis; the urine was normal save for the presence of bile. Since the last confinement also she has developed a xanthomatous condition of the hands, which are studded with little nodules of this condition. All the children had jaundice except the first one, which only lived for an hour; in most of the others jaundice came on at about one week old. The fifth child—the only one breast-fed—is now alive and well, aged twelve years; but all the others died of convulsions. One lived nine months, but the others died in a few weeks. Dr. Tylecote notes the fact that the breast-fed child's survival is a blow to the hypothesis that some maternal toxin excreted through the milk can have been the cause of the fatal jaundice in her offspring. So, too, the fact that xanthoma has been noticed only since the last baby came—about fourteen years after the original attack of jaundice—would seem to negative the supposition that xanthoma in the liver may have been the cause of the trouble. When the patient came under care she was the possessor of a much enlarged liver, and she did not improve.

